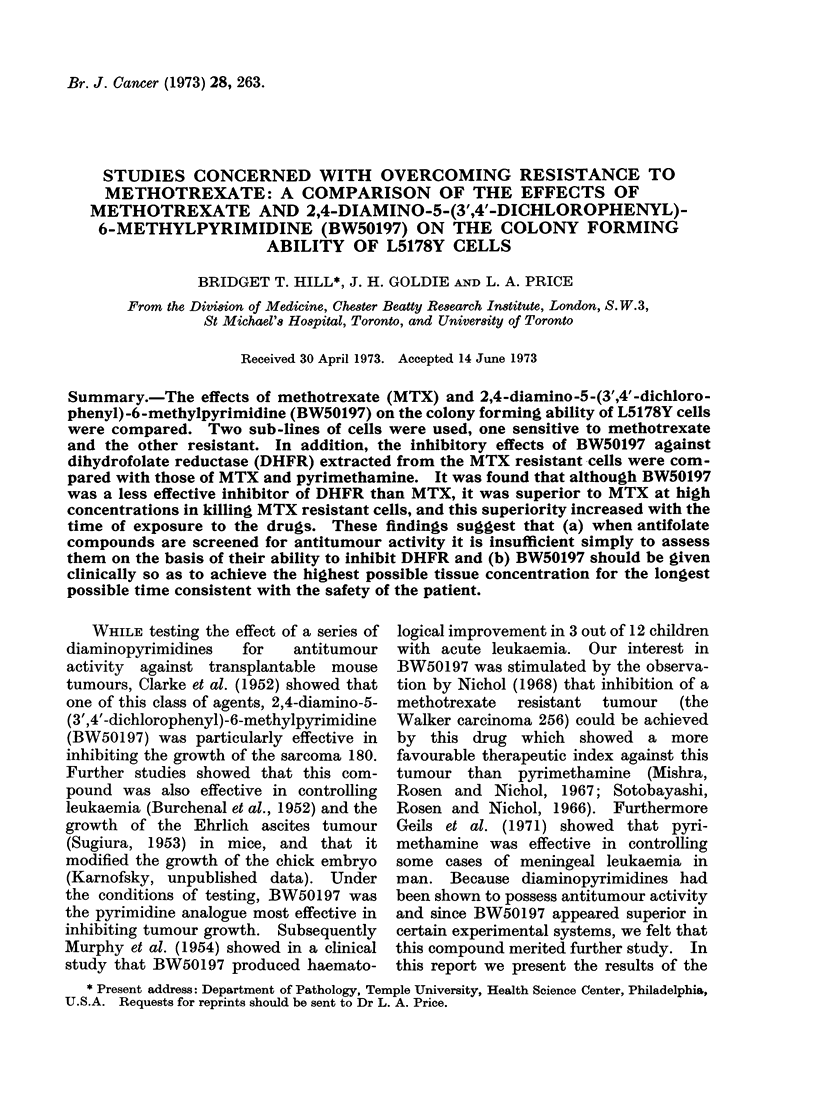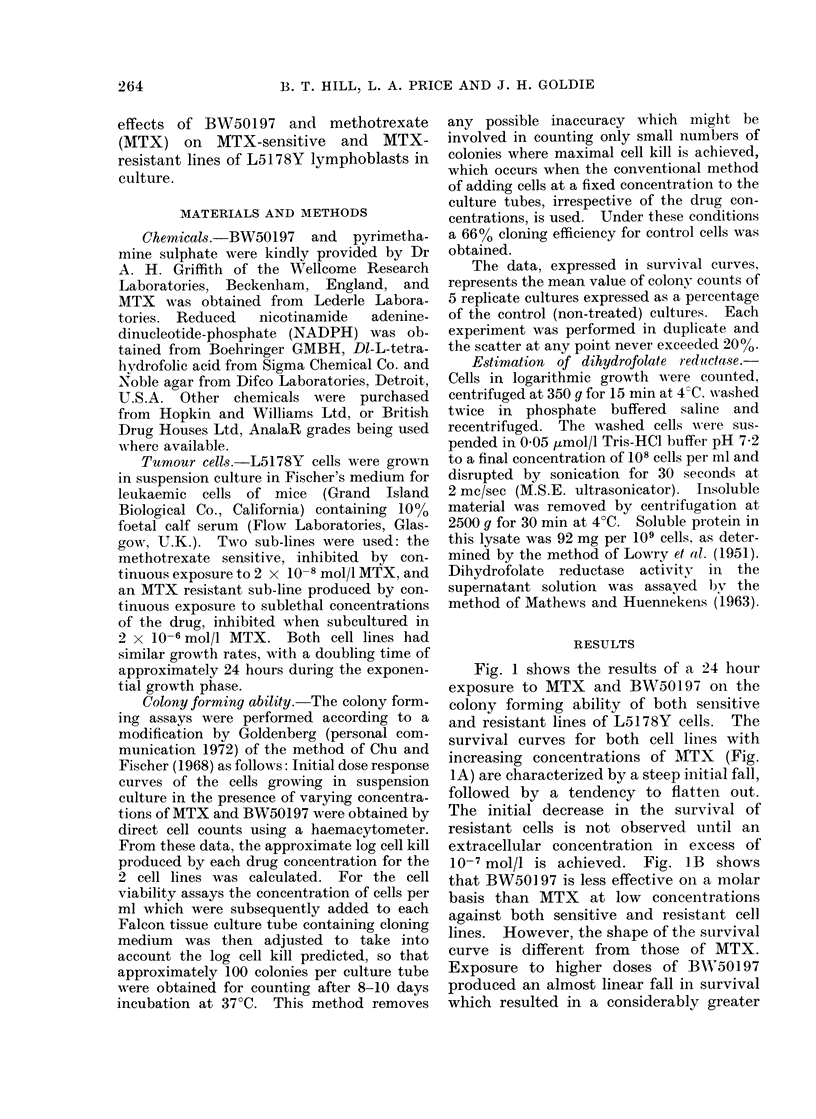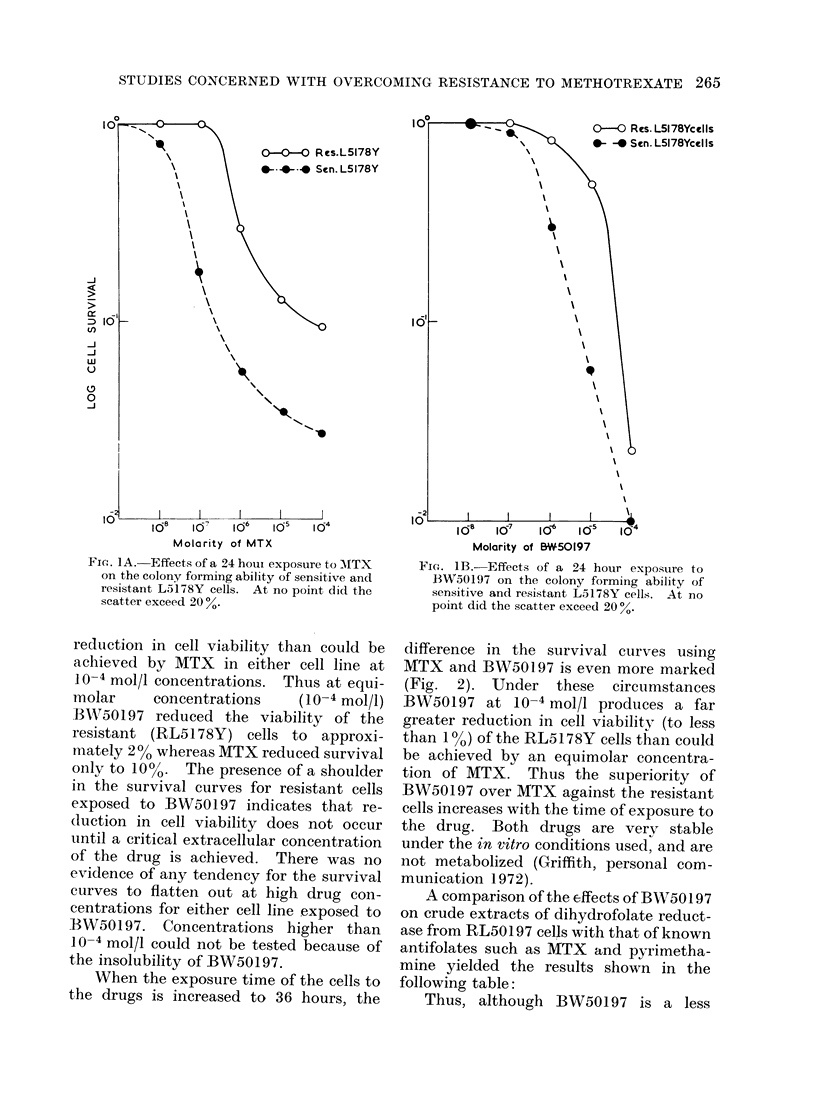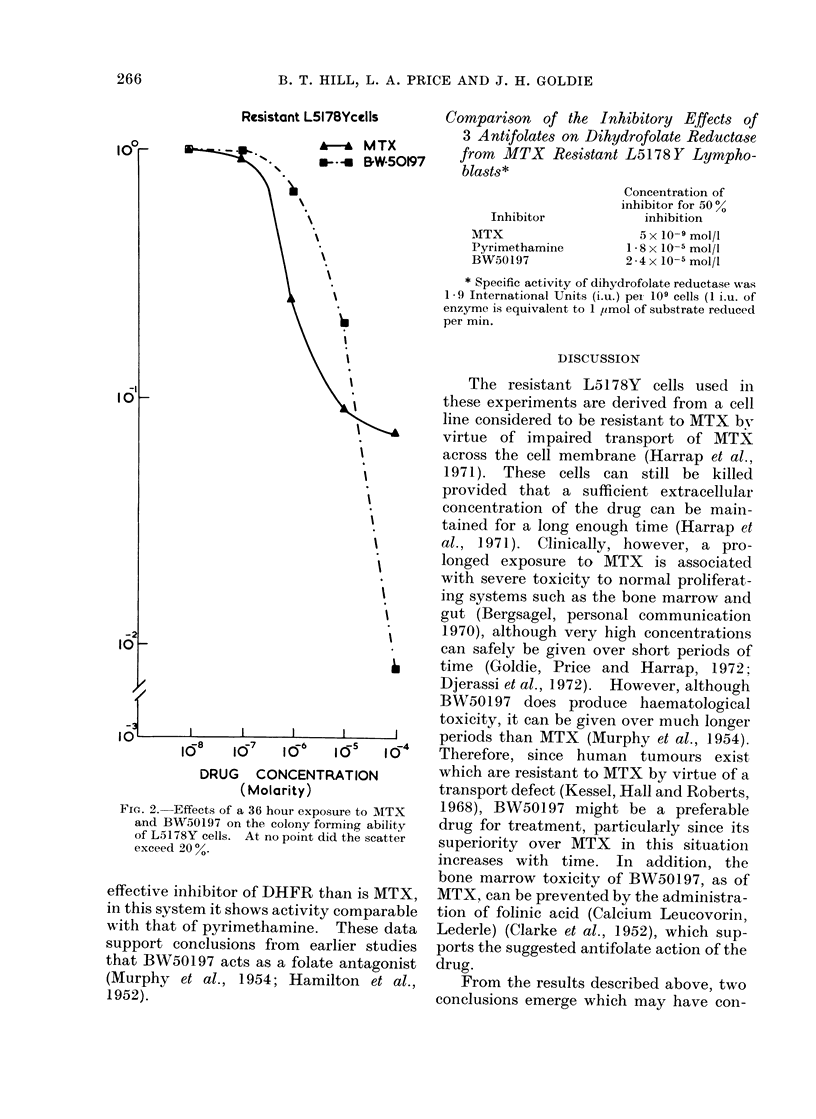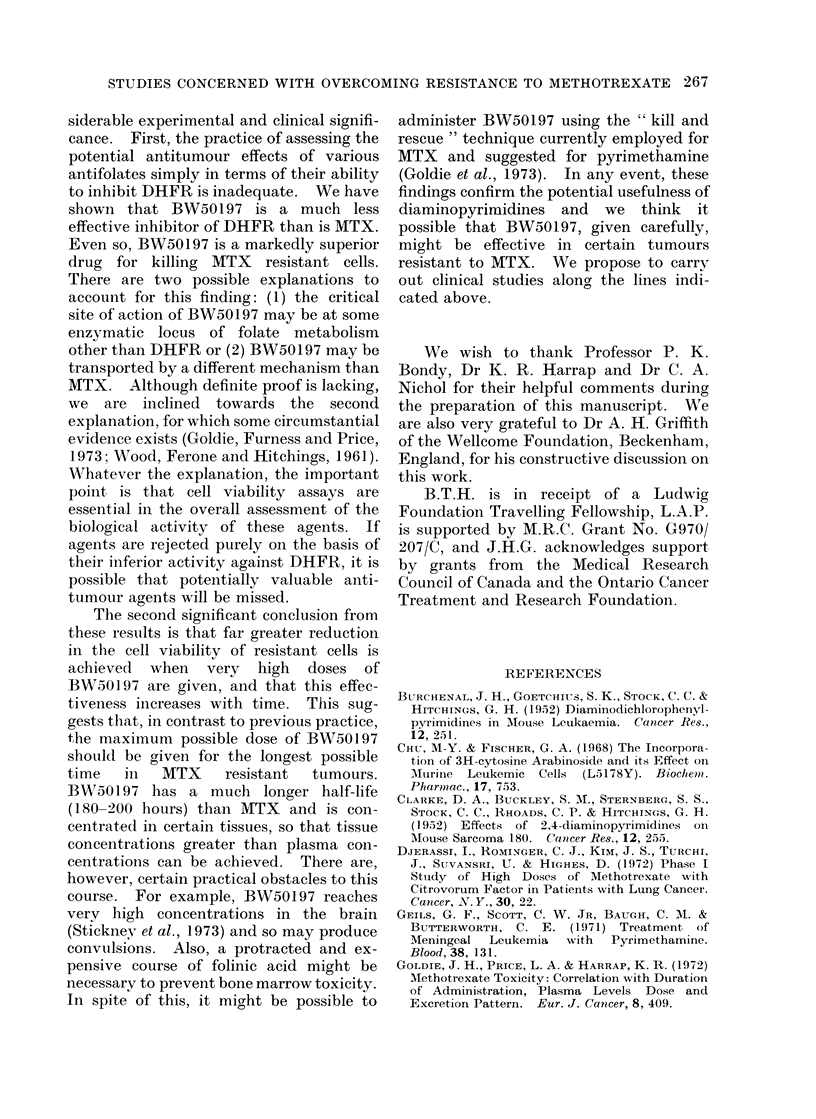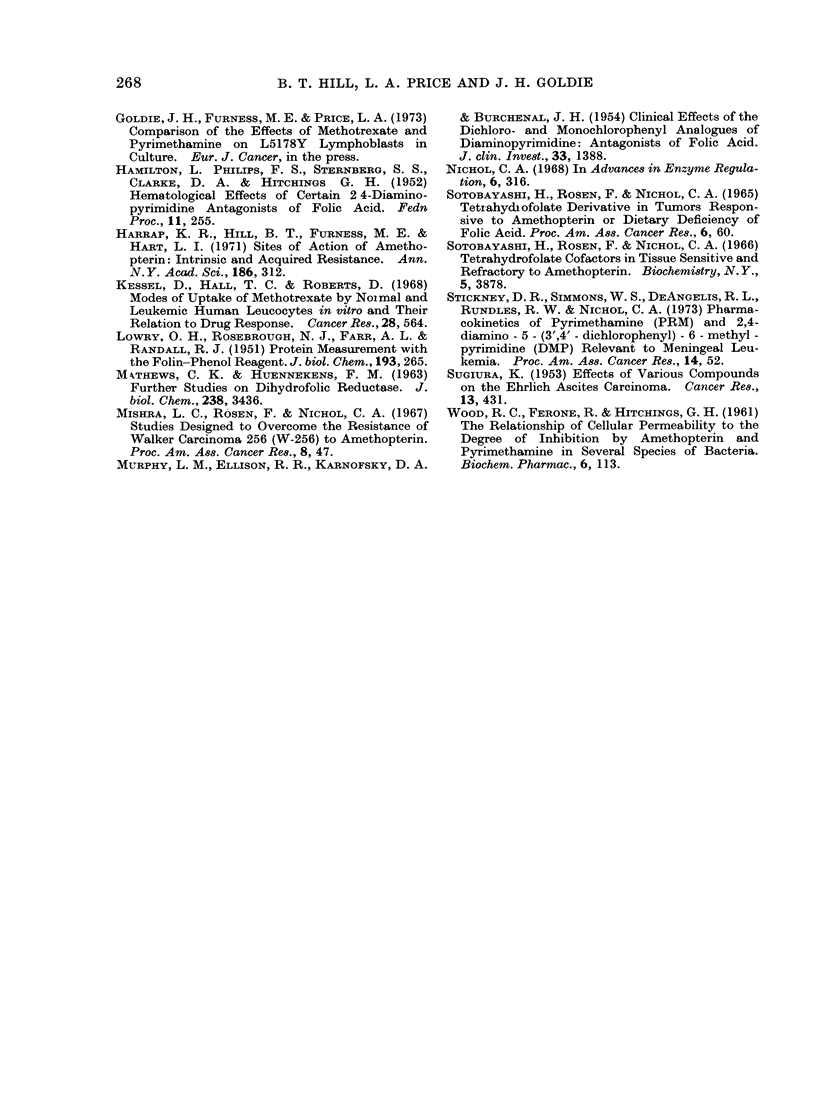# Studies Concerned with Overcoming Resistance to Methotrexate: A Comparison of the Effects of Methotrexate and 2,4-Diamino-5-(3′,4′-Dichlorophenyl)-6-Methylpyrimidine (BW50197) on the Colony Forming Ability of L5178Y Cells

**DOI:** 10.1038/bjc.1973.146

**Published:** 1973-09

**Authors:** Bridget T. Hill, J. H. Goldie, L. A. Price

## Abstract

The effects of methotrexate (MTX) and 2,4-diamino-5-(3′,4′-dichlorophenyl)-6-methylpyrimidine (BW50197) on the colony forming ability of L5178Y cells were compared. Two sub-lines of cells were used, one sensitive to methotrexate and the other resistant. In addition, the inhibitory effects of BW50197 against dihydrofolate reductase (DHFR) extracted from the MTX resistant cells were compared with those of MTX and pyrimethamine. It was found that although BW50197 was a less effective inhibitor of DHFR than MTX, it was superior to MTX at high concentrations in killing MTX resistant cells, and this superiority increased with the time of exposure to the drugs. These findings suggest that (a) when antifolate compounds are screened for antitumour activity it is insufficient simply to assess them on the basis of their ability to inhibit DHFR and (b) BW50197 should be given clinically so as to achieve the highest possible tissue concentration for the longest possible time consistent with the safety of the patient.


*Br. J. Cancer* (1973) **28**, 263.

### STUDIES CONCERNED WITH OVERCOMING RESISTANCE TO METHOTREXATE: A COMPARISON OF THE EFFECTS OF METHOTREXATE AND 2,4-DIAMINO-5-(3′,4′-DICHLOROPHENYL)-6-METHYLPYRIMIDINE (BW50197) ON THE COLONY FORMING ABILITY OF L5178Y CELLS

BRIDGET T. HILL*, J. H. GOLDIE AND L. A. PRICE

*From the Division of Medicine, Chester Beatty Research Institute, London, S.W.3, St Michael's Hospital, Toronto, and University of Toronto*

Received 30 April 1973. Accepted 14 June 1973

**Summary.—The effects of methotrexate (MTX) and 2,4-diamino-5-(3′,4′-dichlorophenyl)-6-methylpyrimidine (BW50197) on the colony forming ability of L5178Y cells were compared. Two sub-lines of cells were used, one sensitive to methotrexate and the other resistant. In addition, the inhibitory effects of BW50197 against dihydrofolate reductase (DHFR) extracted from the MTX resistant cells were compared with those of MTX and pyrimethamine. It was found that although BW50197 was a less effective inhibitor of DHFR than MTX, it was superior to MTX at high concentrations in killing MTX resistant cells, and this superiority increased with the time of exposure to the drugs. These findings suggest that (a) when antifolate compounds are screened for antitumour activity it is insufficient simply to assess them on the basis of their ability to inhibit DHFR and (b) BW50197 should be given clinically so as to achieve the highest possible tissue concentration for the longest possible time consistent with the safety of the patient.**

WHILE testing the effect of a series of diaminopyrimidines for antitumour activity against transplantable mouse tumours, Clarke *et al.* (1952) showed that one of this class of agents, 2,4-diamino-5-(3′,4′-dichlorophenyl)-6-methylpyrimidine (BW50197) was particularly effective in inhibiting the growth of the sarcoma 180. Further studies showed that this compound was also effective in controlling leukaemia (Burchenal *et al.*, 1952) and the growth of the Ehrlich ascites tumour (Sugiura, 1953) in mice, and that it modified the growth of the chick embryo (Karnofsky, unpublished data). Under the conditions of testing, BW50197 was the pyrimidine analogue most effective in inhibiting tumour growth. Subsequently Murphy *et al.* (1954) showed in a clinical study that BW50197 produced haematological improvement in 3 out of 12 children with acute leukaemia. Our interest in BW50197 was stimulated by the observation by Nichol (1968) that inhibition of a methotrexate resistant tumour (the Walker carcinoma 256) could be achieved by this drug which showed a more favourable therapeutic index against this tumour than pyrimethamine (Mishra, Rosen and Nichol, 1967; Sotobayashi, Rosen and Nichol, 1966). Furthermore Geils *et al.* (1971) showed that pyrimethamine was effective in controlling some cases of meningeal leukaemia in man. Because diaminopyrimidines had been shown to possess antitumour activity and since BW50197 appeared superior in certain experimental systems, we felt that this compound merited further study. In this report we present the results of the

* Present address: Department of Pathology, Temple University, Health Science Center, Philadelphia, U.S.A. Requests for reprints should be sent to Dr L. A. Price.

effects of BW50197 and methotrexate (MTX) on MTX-sensitive and MTX-resistant lines of L5178Y lymphoblasts in culture.

### MATERIALS AND METHODS

*Chemicals.*—BW50197 and pyrimethamine sulphate were kindly provided by Dr A. H. Griffith of the Wellcome Research Laboratories, Beckenham, England, and MTX was obtained from Lederle Laboratories. Reduced nicotinamide adenine-dinucleotide-phosphate (NADPH) was obtained from Boehringer GMBH, *Dl*-L-tetra-hydrofolic acid from Sigma Chemical Co. and Noble agar from Difco Laboratories, Detroit, U.S.A. Other chemicals were purchased from Hopkin and Williams Ltd, or British Drug Houses Ltd, AnalaR grades being used where available.

*Tumour cells.*—L5178Y cells were grown in suspension culture in Fischer's medium for leukaemic cells of mice (Grand Island Biological Co., California) containing 10% foetal calf serum (Flow Laboratories, Glasgow, U.K.). Two sub-lines were used: the methotrexate sensitive, inhibited by continuous exposure to $2 \times 10^{-8}$ mol/l MTX, and an MTX resistant sub-line produced by continuous exposure to sublethal concentrations of the drug, inhibited when subcultured in $2 \times 10^{-6}$ mol/l MTX. Both cell lines had similar growth rates, with a doubling time of approximately 24 hours during the exponential growth phase.

*Colony forming ability.*—The colony forming assays were performed according to a modification by Goldenberg (personal communication 1972) of the method of Chu and Fischer (1968) as follows: Initial dose response curves of the cells growing in suspension culture in the presence of varying concentrations of MTX and BW50197 were obtained by direct cell counts using a haemacytometer. From these data, the approximate log cell kill produced by each drug concentration for the 2 cell lines was calculated. For the cell viability assays the concentration of cells per ml which were subsequently added to each Falcon tissue culture tube containing cloning medium was then adjusted to take into account the log cell kill predicted, so that approximately 100 colonies per culture tube were obtained for counting after 8–10 days incubation at 37°C. This method removes any possible inaccuracy which might be involved in counting only small numbers of colonies where maximal cell kill is achieved, which occurs when the conventional method of adding cells at a fixed concentration to the culture tubes, irrespective of the drug concentrations, is used. Under these conditions a 66% cloning efficiency for control cells was obtained.

The data, expressed in survival curves, represents the mean value of colony counts of 5 replicate cultures expressed as a percentage of the control (non-treated) cultures. Each experiment was performed in duplicate and the scatter at any point never exceeded 20%.

*Estimation of dihydrofolate reductase.*—Cells in logarithmic growth were counted, centrifuged at 350 *g* for 15 min at 4°C, washed twice in phosphate buffered saline and recentrifuged. The washed cells were suspended in 0·05 μmol/l Tris-HCl buffer pH 7·2 to a final concentration of $10^8$ cells per ml and disrupted by sonication for 30 seconds at 2 mc/sec (M.S.E. ultrasonicator). Insoluble material was removed by centrifugation at 2500 *g* for 30 min at 4°C. Soluble protein in this lysate was 92 mg per $10^9$ cells, as determined by the method of Lowry *et al.* (1951). Dihydrofolate reductase activity in the supernatant solution was assayed by the method of Mathews and Huennekens (1963).

### RESULTS

Fig. 1 shows the results of a 24 hour exposure to MTX and BW50197 on the colony forming ability of both sensitive and resistant lines of L5178Y cells. The survival curves for both cell lines with increasing concentrations of MTX (Fig. 1A) are characterized by a steep initial fall, followed by a tendency to flatten out. The initial decrease in the survival of resistant cells is not observed until an extracellular concentration in excess of $10^{-7}$ mol/l is achieved. Fig. 1B shows that BW50197 is less effective on a molar basis than MTX at low concentrations against both sensitive and resistant cell lines. However, the shape of the survival curve is different from those of MTX. Exposure to higher doses of BW50197 produced an almost linear fall in survival which resulted in a considerably greater

FIG. 1A.—Effects of a 24 hour exposure to MTX on the colony forming ability of sensitive and resistant L5178Y cells. At no point did the scatter exceed 20%.

FIG. 1B.—Effects of a 24 hour exposure to BW50197 on the colony forming ability of sensitive and resistant L5178Y cells. At no point did the scatter exceed 20%.

reduction in cell viability than could be achieved by MTX in either cell line at $10^{-4}$ mol/l concentrations. Thus at equimolar concentrations ($10^{-4}$ mol/l) BW50197 reduced the viability of the resistant (RL5178Y) cells to approximately 2% whereas MTX reduced survival only to 10%. The presence of a shoulder in the survival curves for resistant cells exposed to BW50197 indicates that reduction in cell viability does not occur until a critical extracellular concentration of the drug is achieved. There was no evidence of any tendency for the survival curves to flatten out at high drug concentrations for either cell line exposed to BW50197. Concentrations higher than $10^{-4}$ mol/l could not be tested because of the insolubility of BW50197.

When the exposure time of the cells to the drugs is increased to 36 hours, the difference in the survival curves using MTX and BW50197 is even more marked (Fig. 2). Under these circumstances BW50197 at $10^{-4}$ mol/l produces a far greater reduction in cell viability (to less than 1%) of the RL5178Y cells than could be achieved by an equimolar concentration of MTX. Thus the superiority of BW50197 over MTX against the resistant cells increases with the time of exposure to the drug. Both drugs are very stable under the *in vitro* conditions used, and are not metabolized (Griffith, personal communication 1972).

A comparison of the effects of BW50197 on crude extracts of dihydrofolate reductase from RL50197 cells with that of known antifolates such as MTX and pyrimethamine yielded the results shown in the following table:

Thus, although BW50197 is a less

FIG. 2.—Effects of a 36 hour exposure to MTX and BW50197 on the colony forming ability of L5178Y cells. At no point did the scatter exceed 20%.

effective inhibitor of DHFR than is MTX, in this system it shows activity comparable with that of pyrimethamine. These data support conclusions from earlier studies that BW50197 acts as a folate antagonist (Murphy *et al.*, 1954; Hamilton *et al.*, 1952).

*Comparison of the Inhibitory Effects of 3 Antifolates on Dihydrofolate Reductase from MTX Resistant L5178Y Lymphoblasts**

| Inhibitor | Concentration of inhibitor for 50% inhibition |
|---|---|
| MTX | $5 \times 10^{-9}$ mol/l |
| Pyrimethamine | $1 \cdot 8 \times 10^{-5}$ mol/l |
| BW50197 | $2 \cdot 4 \times 10^{-5}$ mol/l |

* Specific activity of dihydrofolate reductase was 1·9 International Units (i.u.) per $10^9$ cells (1 i.u. of enzyme is equivalent to 1 μmol of substrate reduced per min.

### DISCUSSION

The resistant L5178Y cells used in these experiments are derived from a cell line considered to be resistant to MTX by virtue of impaired transport of MTX across the cell membrane (Harrap *et al.*, 1971). These cells can still be killed provided that a sufficient extracellular concentration of the drug can be maintained for a long enough time (Harrap *et al.*, 1971). Clinically, however, a prolonged exposure to MTX is associated with severe toxicity to normal proliferating systems such as the bone marrow and gut (Bergsagel, personal communication 1970), although very high concentrations can safely be given over short periods of time (Goldie, Price and Harrap, 1972; Djerassi *et al.*, 1972). However, although BW50197 does produce haematological toxicity, it can be given over much longer periods than MTX (Murphy *et al.*, 1954). Therefore, since human tumours exist which are resistant to MTX by virtue of a transport defect (Kessel, Hall and Roberts, 1968), BW50197 might be a preferable drug for treatment, particularly since its superiority over MTX in this situation increases with time. In addition, the bone marrow toxicity of BW50197, as of MTX, can be prevented by the administration of folinic acid (Calcium Leucovorin, Lederle) (Clarke *et al.*, 1952), which supports the suggested antifolate action of the drug.

From the results described above, two conclusions emerge which may have considerable experimental and clinical significance. First, the practice of assessing the potential antitumour effects of various antifolates simply in terms of their ability to inhibit DHFR is inadequate. We have shown that BW50197 is a much less effective inhibitor of DHFR than is MTX. Even so, BW50197 is a markedly superior drug for killing MTX resistant cells. There are two possible explanations to account for this finding: (1) the critical site of action of BW50197 may be at some enzymatic locus of folate metabolism other than DHFR or (2) BW50197 may be transported by a different mechanism than MTX. Although definite proof is lacking, we are inclined towards the second explanation, for which some circumstantial evidence exists (Goldie, Furness and Price, 1973; Wood, Ferone and Hitchings, 1961). Whatever the explanation, the important point is that cell viability assays are essential in the overall assessment of the biological activity of these agents. If agents are rejected purely on the basis of their inferior activity against DHFR, it is possible that potentially valuable antitumour agents will be missed.

The second significant conclusion from these results is that far greater reduction in the cell viability of resistant cells is achieved when very high doses of BW50197 are given, and that this effectiveness increases with time. This suggests that, in contrast to previous practice, the maximum possible dose of BW50197 should be given for the longest possible time in MTX resistant tumours. BW50197 has a much longer half-life (180–200 hours) than MTX and is concentrated in certain tissues, so that tissue concentrations greater than plasma concentrations can be achieved. There are, however, certain practical obstacles to this course. For example, BW50197 reaches very high concentrations in the brain (Stickney *et al.*, 1973) and so may produce convulsions. Also, a protracted and expensive course of folinic acid might be necessary to prevent bone marrow toxicity. In spite of this, it might be possible to administer BW50197 using the " kill and rescue " technique currently employed for MTX and suggested for pyrimethamine (Goldie *et al.*, 1973). In any event, these findings confirm the potential usefulness of diaminopyrimidines and we think it possible that BW50197, given carefully, might be effective in certain tumours resistant to MTX. We propose to carry out clinical studies along the lines indicated above.

We wish to thank Professor P. K. Bondy, Dr K. R. Harrap and Dr C. A. Nichol for their helpful comments during the preparation of this manuscript. We are also very grateful to Dr A. H. Griffith of the Wellcome Foundation, Beckenham, England, for his constructive discussion on this work.

B.T.H. is in receipt of a Ludwig Foundation Travelling Fellowship, L.A.P. is supported by M.R.C. Grant No. G970/207/C, and J.H.G. acknowledges support by grants from the Medical Research Council of Canada and the Ontario Cancer Treatment and Research Foundation.

### REFERENCES

BURCHENAL, J. H., GOETCHIUS, S. K., STOCK, C. C. & HITCHINGS, G. H. (1952) Diaminodichlorophenylpyrimidines in Mouse Leukaemia. *Cancer Res.*, **12**, 251.

CHU, M-Y. & FISCHER, G. A. (1968) The Incorporation of 3H-cytosine Arabinoside and its Effect on Murine Leukemic Cells (L5178Y). *Biochem. Pharmac.*, **17**, 753.

CLARKE, D. A., BUCKLEY, S. M., STERNBERG, S. S., STOCK, C. C., RHOADS, C. P. & HITCHINGS, G. H. (1952) Effects of 2,4-diaminopyrimidines on Mouse Sarcoma 180. *Cancer Res.*, **12**, 255.

DJERASSI, I., ROMINGER, C. J., KIM, J. S., TURCHI, J., SUVANSRI, U. & HIGHES, D. (1972) Phase I Study of High Doses of Methotrexate with Citrovorum Factor in Patients with Lung Cancer. *Cancer, N.Y.*, **30**, 22.

GEILS, G. F., SCOTT, C. W. JR, BAUGH, C. M. & BUTTERWORTH, C. E. (1971) Treatment of Meningeal Leukemia with Pyrimethamine. *Blood*, **38**, 131.

GOLDIE, J. H., PRICE, L. A. & HARRAP, K. R. (1972) Methotrexate Toxicity: Correlation with Duration of Administration, Plasma Levels Dose and Excretion Pattern. *Eur. J. Cancer*, **8**, 409.

GOLDIE, J. H., FURNESS, M. E. & PRICE, L. A. (1973) Comparison of the Effects of Methotrexate and Pyrimethamine on L5178Y Lymphoblasts in Culture. *Eur. J. Cancer*, in the press.

HAMILTON, L. PHILIPS, F. S., STERNBERG, S. S., CLARKE, D. A. & HITCHINGS G. H. (1952) Hematological Effects of Certain 2 4-Diaminopyrimidine Antagonists of Folic Acid. *Fedn Proc.*, **11**, 255.

HARRAP, K. R., HILL, B. T., FURNESS, M. E. & HART, L. I. (1971) Sites of Action of Amethopterin: Intrinsic and Acquired Resistance. *Ann. N.Y. Acad. Sci.*, **186**, 312.

KESSEL, D., HALL, T. C. & ROBERTS, D. (1968) Modes of Uptake of Methotrexate by Noimal and Leukemic Human Leucocytes *in vitro* and Their Relation to Drug Response. *Cancer Res.*, **28**, 564.

LOWRY, O. H., ROSEBROUGH, N. J., FARR, A. L. & RANDALL, R. J. (1951) Protein Measurement with the Folin–Phenol Reagent. *J. biol. Chem.*, **193**, 265.

MATHEWS, C. K. & HUENNEKENS, F. M. (1963) Further Studies on Dihydrofolic Reductase. *J. biol. Chem.*, **238**, 3436.

MISHRA, L. C., ROSEN, F. & NICHOL, C. A. (1967) Studies Designed to Overcome the Resistance of Walker Carcinoma 256 (W-256) to Amethopterin. *Proc. Am. Ass. Cancer Res.*, **8**, 47.

MURPHY, L. M., ELLISON, R. R., KARNOFSKY, D. A. & BURCHENAL, J. H. (1954) Clinical Effects of the Dichloro- and Monochlorophenyl Analogues of Diaminopyrimidine: Antagonists of Folic Acid. *J. clin. Invest.*, **33**, 1388.

NICHOL, C. A. (1968) In *Advances in Enzyme Regulation*, **6**, 316.

SOTOBAYASHI, H., ROSEN, F. & NICHOL, C. A. (1965) Tetiahydiofolate Derivative in Tumors Responsive to Amethopterin or Dietary Deficiency of Folic Acid. *Proc. Am. Ass. Cancer Res.*, **6**, 60.

SOTOBAYASHI, H., ROSEN, F. & NICHOL, C. A. (1966) Tetrahydrofolate Cofactors in Tissue Sensitive and Refractory to Amethopterin. *Biochemistry, N.Y.*, **5**, 3878.

STICKNEY, D. R., SIMMONS, W. S., DEANGELIS, R. L., RUNDLES, R. W. & NICHOL, C. A. (1973) Pharmacokinetics of Pyrimethamine (PRM) and 2,4-diamino - 5 - (3′,4′ - dichlorophenyl) - 6 - methyl - pyrimidine (DMP) Relevant to Meningeal Leukemia. *Proc. Am. Ass. Cancer Res.*, **14**, 52.

SUGIURA, K. (1953) Effects of Various Compounds on the Ehrlich Ascites Carcinoma. *Cancer Res.*, **13**, 431.

WOOD, R. C., FERONE, R. & HITCHINGS, G. H. (1961) The Relationship of Cellular Permeability to the Degree of Inhibition by Amethopterin and Pyrimethamine in Several Species of Bacteria. *Biochem. Pharmac.*, **6**, 113.